# Absolute protein quantitation of the mouse macrophage Toll-like receptor and chemotaxis pathways

**DOI:** 10.1038/s41597-022-01612-y

**Published:** 2022-08-12

**Authors:** Nathan P. Manes, Jessica M. Calzola, Pauline R. Kaplan, Iain D. C. Fraser, Ronald N. Germain, Martin Meier-Schellersheim, Aleksandra Nita-Lazar

**Affiliations:** Laboratory of Immune System Biology, National Institute of Allergy and Infectious Diseases, National Institutes of Health, Bethesda, Maryland USA

**Keywords:** Toll-like receptors, Protein-protein interaction networks

## Abstract

The Toll-like receptor (TLR) and chemotaxis pathways are key components of the innate immune system. Subtle variation in the concentration, timing, and molecular structure of the ligands are known to affect downstream signaling and the resulting immune response. Computational modeling and simulation at the molecular interaction level can be used to study complex biological pathways, but such simulations require protein concentration values as model parameters. Here we report the development and application of targeted mass spectrometry assays to measure the absolute abundance of proteins of the mouse macrophage Toll-like receptor 4 (TLR4) and chemotaxis pathways. Two peptides per protein were quantified, if possible. The protein abundance values ranged from 1,332 to 227,000,000 copies per cell. They moderately correlated with transcript abundance values from a previously published mouse macrophage RNA-seq dataset, and these two datasets were combined to make proteome-wide abundance estimates. The datasets produced during this investigation can be used for pathway modeling and simulation, as well as for other studies of the TLR and chemotaxis pathways.

## Abstract (summary)

**Table:** 

Measurement(s)	molecules per cell
Technology Type(s)	nanoflow high-performance liquid chromatography-electrospray ionisation tandem mass spectrometry
Sample Characteristic - Organism	Mus musculus

## Background & Summary

Eukaryotic cells contain intricate pathways to sense and respond to threats by pathogens^1^. The innate immune response is the initial defense against invasion by pathogens, and it is activated upon association of pathogen-associated molecular patterns (PAMPs) with host pattern recognition receptors (PRRs). Bacterial lipopolysaccharide (LPS) binding to and activating Toll-like receptor 4 (TLR4) is a well-characterized PAMP-PRR interaction. PRR pathway activation generally results in upregulation of cytokine synthesis and release. A special class of cytokines called chemokines (chemotactic cytokines) bind and activate leukocyte chemokine receptors, which cause chemotaxis (directed cellular movement) along chemokine concentration gradients^2–5^. Chemotaxis requires highly synchronized cellular mechanisms including protrusion, adhesion, and force generation at the leading end, and de-adhesion and contraction at the trailing end.

The PRR and chemotaxis pathways have a major role in a wide range of innate immune physiological and pathophysiological processes. Due to the complexity of PRR and chemotaxis pathways, accurate modeling and simulation at a molecular, mechanistic level can be an important contribution to fully understand these pathways and to aid the development of therapeutics to target related disorders. To address the technical challenges associated with developing models of complex signaling pathways, rule-based spatiotemporal modeling and simulation approaches can be used. However, they require model parameters such as molecular concentrations and interaction rates^6,7^. Measuring the *in vivo* reaction rates of a biological pathway remains challenging, but they can be predicted using molecular simulation^8,9^.

Recently, targeted proteomics using liquid chromatography coupled to mass spectrometry (LC-MS) has developed into a powerful tool to measure the absolute abundance (*e.g*., copies/cell) of proteins^10^. Targeted proteomics has been used to study proteogenomics (including splice isoforms, single amino acid polymorphisms, and other variants), posttranslational modifications, kinomics, protein turnover, protein conformation, protein-protein interaction, protein interactions with other molecules, protein-protein subcellular proximity, and metabolic and signaling pathways^10^. In this report, we describe how we have used targeted LC-MS to quantitate proteins of the mouse macrophage TLR4 and chemotaxis pathways. Using these data and a previously published RNA-seq dataset, we calculated proteome-wide abundance estimates. These data can be used for biological pathway modeling and simulation, as well as for other systems biology research.

## Methods

### Outline of the development of the targeted LC-MS assays

Target proteins were selected for absolute quantitation to study the chemotaxis and TLR pathways in mouse macrophages. Tryptic target peptides were selected, and crude unlabeled target peptide mixes were purchased and analyzed by using a triple quadrupole (QqQ) mass spectrometer to perform shotgun data-dependent acquisition (DDA) LC-MS(/MS). The resulting spectra were used to make spectral libraries and selected reaction monitoring (SRM) LC-MS assays. The LC-SRM assays were used to analyze the crude target peptide mixes, and an assay was discarded if it failed to produce confident identifications. The successful LC-SRM assays were used to analyze RAW264.7 cell preparations, and an assay was discarded if it failed to produce confident identifications. For each target protein, at least two target peptide LC-SRM assays were developed, if possible, to enable at least two independent measurements of the absolute abundance of each target protein. Multiple rounds of LC-SRM assay development were necessary. For the chemotaxis pathway, this has been described previously^2,11^.

Heavy isotope-labeled, purified, quantitated peptide standards were purchased for targeted LC-MS assays to measure the absolute abundance of the target proteins. Concurrently, our laboratory acquired a new LC-MS system: an UltiMate 3000 nanoLC coupled to a Q Exactive HF (QEHF) mass spectrometer (Thermo Fisher Scientific Inc., Waltham, MA). This system can perform parallel reaction monitoring (PRM) LC-MS. Compared to our LC-QqQ system, our LC-QEHF system is capable of far higher mass accuracy and resolution, which greatly reduced interference by coeluting analytes, thus decreasing false positive peptide identifications and increasing quantitation accuracy. In addition, our LC-QEHF system is capable of advanced LC scheduling capabilities that our LC-QqQ system lacks, enabling a sample throughput of approximately 5-fold greater.

Consequently, LC-PRM assays were developed using the heavy isotope-labeled, purified, quantitated peptide standard mixes. To this end, these peptide mixes were prepared for LC-MS as described below and analyzed using both shotgun and targeted LC-QEHF-DDA-MS(/MS). The resulting spectra were used to make spectral libraries and LC-PRM assays. Subsequently, LC-PRM of these peptide mixes was used to develop scheduled LC-PRM assays, and these were used to measure the absolute abundance of the target proteins. The experimental procedures are described in detail below.

### Target protein and peptide selection

Proteins of the mouse chemotaxis and TLR4 signaling pathways were targeted for absolute quantitation by LC-MS. The chemotaxis pathway target proteins are described in our earlier publication^2^. An extensive literature review was performed to construct a mechanistic network of mouse pattern recognition receptor pathways^1^, and the principal signaling interactions of the TLR4 pathway were manually selected. A small number of other target proteins were included (housekeeping proteins for normalization across samples, and *Photinus pyralis* (firefly) luciferase to function as a quantitated internal protein standard). All protein sequences were retrieved from UniProt^12^. The target proteins are tabulated in the file “Targeted Proteins.xlsx” available at Panorama Public^13^.

The target peptide selection criteria were similar to those in our previously published protocol^11^. Peptides were manually selected to be both proteotypic (*i.e*., efficiently identified and quantitated by LC-MS), and quantotypic (the peptide quantity is an accurate measurement of the target protein quantity). An *ad hoc* score was used to rank and select the best candidate target peptides. This was aided by using Skyline^14^ and PeptidePicker^15^. The selection criteria were:

1. The peptide must be fully tryptic with no missing cleavages. Avoid internal KP and RP sites as trypsin might slowly digest these^16^.
2. Avoid neighboring trypsin cleavage sites (*e.g*., AAK.R.AA) because these might be digested relatively slowly^16^.
3. Preferentially, tryptic proteolysis produces exactly one copy of the target peptide per copy of the target protein (consider Ile/Leu substitution because these peptides perform nearly identically during LC-MS(/MS)).
4. Preferentially, the peptide length is 5–20 amino acids. Shorter peptides will probably not be unique and will produce few transitions. Longer peptides are difficult to synthesize and thus are relatively expensive.
5. Avoid Cys and Met (oxidation), Asn and Gln (deamidation), and amino-terminal Gln (formation of pyroglutamate). Avoid protein N/C-termini because they are prone to PTMs (*e.g*., C-term amidation and N-term methionine loss and acetylation). Avoid any other covalent modifications including PTMs and chemical artifacts (specifically, the PTMs annotated in UniProt were considered) (also check that the trypsin sites are unaffected).
6. Avoid a peptide if it corresponds to a natural genetic variant (specifically, the variants annotated in UniProt) (also check that the trypsin sites are unaffected).
7. Preferentially, the peptide would be useful for assays of orthologs (specifically, human orthologs were considered) (check that the trypsin sites are still present and consider Ile/Leu substitution).
8. Require that the peptide be unique to the target protein within the biological samples (consider Ile/Leu substitutions). If the peptide is not unique, consider using it if it is unique to a small set of closely related homologues (to assay the combined abundance). If a proteome of the biological samples is available (translated RNA-seq data of mouse macrophages were used^2^), it might be a more accurate search-space than the whole proteome of the species.
9. If splice isoform data are available (specifically, RNA-seq data^2^ and annotated splice isoforms in UniProt), consider the usefulness of the target peptide against the most abundant mRNA splice isoform(s).
10. The target peptide must be proteotypic. Avoid peptides that produce a small number of transitions (<6). Avoid highly hydrophobic or hydrophilic peptides. Use LC-MS proteomics data to determine which peptides produce the most intense precursor ions and produce highly confident peptide-spectrum matches (PSMs). Spectra from The Global Proteome Machine^17^ and the National Institute of Standards and Technology^18^ online databases were used. In addition, spectra from our own laboratory were used^19–21^.

### Preparation of the peptide standards

Custom peptide standards were purchased from JPT Peptide Technologies GmbH (Berlin, Germany) and Thermo Fisher Scientific Inc. In total, 851 crude light (*i.e*., not labeled with stable isotopes) peptides were purchased for qualitative analyses, and 279 purified quantitated heavy-labeled (^13^C_6_^15^N_4_ Arg, ^13^C_6_^15^N_2_ Lys) internal peptide standards were purchased for quantitative analyses. The target peptides are tabulated in the file “Targeted Peptides.xlsx” available at Panorama Public^13^. Of the 279 quantitated peptides, 29 are phosphopeptides for phosphoprotein quantitation.

Most of the internal peptide standards were quantitated using UV absorption (at 350 and/or 428 nm) of a carboxy-terminal trypsin-cleavable “quantification tag” (Qtag)^22^. The Qtag is a tetrapeptide which includes a nitrotyrosine residue which is absorbent at 350 and 428 nm. Absorbance by natural amino acids is insignificant at 350 and 428 nm, and the Qtag was used to accurately measure the absolute abundance of these internal peptide standards. The remaining internal peptide standards were quantitated using amino acid analysis (AAA). When possible, each peptide was flanked by leading and trailing regions (each was three amino acids in length) to mimic the cleavage site of the target protein. In contrast to the internal peptide standards (used for absolute abundance measurements), the unlabeled crude peptides (used for LC-MS assay development) were fully tryptic and did not require trypsin digestion.

The quantitated peptide standards were dissolved in 20% v/v acetonitrile (ACN), vortexed for 2.5 min, and bath sonicated for 5 min at room temperature. Peptides were pooled and concentrated in a SpeedVac at 40 °C. The final condition of each mixture of peptides was 4 µM (of each peptide) in 20% v/v ACN. This procedure was performed in duplicate (*i.e*., a total of two JPT/Thermo aliquots of each peptide were independently prepared), and then mixed. The crude peptide standards were prepared in the same way (except that the peptide abundance was unknown).

### Cell culture

RAW264.7 (American Type Culture Collection TIB-71) mouse macrophages and immortalized mouse macrophage (IMM) cells^23^ were cultured in Complete Medium: Dulbecco’s Modified Eagle’s medium (DMEM; 4.5 g/L glucose; 4 mM L-glutamine), 20 mM HEPES, 1 mM sodium pyruvate, and 10% v/v fetal bovine serum (not heat inactivated). Cells were counted (and cell diameters were measured) using a Cellometer Auto T4 (Nexcelom Bioscience LLC, Lawrence, MA), and trypan blue staining was used to measure viability.

Bone marrow-derived macrophages (BMDMs) were prepared using a protocol very similar to two previously published protocols^24,25^. All procedures were approved by the NIAID Animal Care and Use Committee (NIH). C57BL/6 J mice (male, 19 wk old, The Jackson Laboratory, Bar Harbor, ME) were euthanized using CO_2_ followed by cervical dislocation. Femurs and tibias were isolated and placed in ice-cold DMEM. One bone at a time, the ends were cut off using surgical scissors, and the bone marrow was pushed out using a syringe loaded with DMEM and attached to a 27 gauge needle. The cell suspension was gently pipetted to break up clumps and strained using a CytoStrainer cell strainer (70 micron, Alkali Scientific Inc., Fort Lauderdale, FL). The cells were pelleted using centrifugation and suspended in ACK lysing buffer (Quality Biological, Gaithersburg, MD) to lyse red blood cells. The cells were diluted in DMEM to stop the reaction, pelleted using centrifugation, and suspended and differentiated for six days in Complete Medium supplemented with 60 ng/ml mouse MCSF (R&D Systems Inc, Minneapolis, MN). The BMDMs were used for experiments within 24 h. The cells were counted as described above.

### Sample preparation for LC-MS

These procedures are based on our published protocol^11^. The cells were lysed and homogenized using a Bioruptor Plus (20 min with power set to “High”, Diagenode Inc., Denville, NJ) in Lysis Buffer: 8 M urea, 100 mM HEPES∙NaOH, pH 8, 10 µM bestatin, 10 µM pepstatin A, 1x Halt phosphatase inhibitor cocktail (provided as a 100x solution; Thermo Fisher Scientific Inc.). Cell lysate protein concentrations were measured using a bicinchoninic acid (BCA) assay kit (Thermo Fisher Scientific Inc.). The median protein mass per BMDM cell was 230 pg/cell. To confirm that the unstimulated BMDMs did not contain protein phosphorylation indicative of TLR activation, western blots were performed for phospho-JNK and phospho-ERK1/2 (cat#s 4668 s, 4370 S, Cell Signaling Technology Inc., Danvers, MA) (LPS-stimulated BMDMs were used as a positive control: 30 min stimulation with 10 nM Kdo2-Lipid A, Avanti Polar Lipids, Birmingham, AL).

A set of eleven samples were prepared for LC-PRM to measure the copies per BMDM cell of the target proteins. Two BMDM biological replicates were prepared (using one mouse each). Either 0 μg or 50 μg (protein mass) of a BMDM homogenate was transferred to a microcentrifuge tube containing Lysis Buffer such that the final volume was 45.8 μl. One pmol of Photinus pyralis (firefly) luciferase (Sigma-Aldrich, now Millipore Sigma, Merck KGaA, Darmstadt, Germany) was added to each sample to function as a quantitated internal protein standard. Either 0, 50, 500, or 5000 fmol (of each peptide) of the quantitated heavy labeled peptide standards mixture was added. Consequently, each sample volume was 60 μl.

DTT was added to each sample (0.6 µl of 1 M DTT; final concentration = 10 mM DTT), and the samples were incubated at 60 °C for 30 min to reduce sample protein cystines. Iodoacetamide was added to each sample (6.06 µl of 500 mM iodoacetamide, 500 mM HEPES∙NaOH pH 8; final concentration = 50 mM iodoacetamide), and the samples were incubated at room temperature for 20 min in darkness to alkylate sample protein cystines. Each sample was diluted by adding 411.34 μl of 100 mM HEPES∙NaOH pH 8 so that the final urea concentration was ≤1 M (trypsin works poorly at >1 M urea).

For each of the samples that did not contain BMDM lysate (“B0-1”, “B0-2”, and “B0-3”), 1 µl of 0.5 µg/µl sequencing grade modified trypsin (Promega Corp., Madison, WI) was added, and they were incubated at 37 °C for two hours. For each of the other eight samples, 1:50 trypsin:sample (protein w:w) was added, and they were incubated at 37 °C for 16 hours. To halt the reactions, 120 µl of 5% v/v formic acid (FA) was added to each sample.

The digests were microcentrifuged at 10,000 × g for 20 min at room temperature to pellet anything that might clog a Sep-Pak column. Each sample underwent solid-phase extraction (SPE) using a Sep-Pak C-18 SPE column (1 ml, 100 mg C-18 media, Waters Corp., Milford, MA; Mobile Phase A = 0.1% v/v FA, Mobile Phase B = 0.1% v/v FA, 80% v/v ACN). The Sep-Pak eluates were concentrated in a SpeedVac at 35 °C to a volume of 80 µl. To ensure that the ACN was completely removed, 100 µl of 0.1% v/v FA, 2% v/v ACN was added to each sample, and they were concentrated in a SpeedVac at 35 °C to a volume of 50 µl. For each sample, 200 µl of 0.1% v/v FA, 2% v/v ACN, 25 nM Pierce LC Retention Time peptide standards (Thermo Fisher Scientific Inc.) was added.

In addition to the above eleven sample preparations, additional samples were prepared using the same protocol. These were preparations of the heavy-labeled peptides (alone), RAW264.7 cells, and IMM cells. These samples were analyzed to support LC-MS assay development (described below).

### Mass spectrometry

SRM LC-MS was performed using a 1200 series nanoLC (Agilent Technologies, Santa Clara, CA) coupled to a TSQ Vantage QqQ mass spectrometer (Thermo Fisher Scientific Inc.). The samples were pumped directly onto a resolving column consisting of coated silica capillary (50 µm ID) with a laser-pulled tip (Laser Based Micropipette Puller, Sutter Instrument Co., Novato, CA) packed using a pressure cell (column length = 15 cm) with Magic C18AQ resin (5 µm diameter, 200 A pores, Bruker Corp., Billerica, MA). Analytes were separated using a 60 min linear gradient (0 – 40% Mobile Phase B; Mobile Phase A = 0.1% v/v FA; Mobile Phase B = 0.1% v/v FA in ACN; flow rate = 200 nl/min). Analytes were electrosprayed at 1.8 kV into the QqQ MS (Q1 isolation width = 0.7 m/z; q2 argon pressure = 1.5 mTorr; Q3 isolation width = 0.7 m/z; dwell time = 10 ms; collision energy settings described previously^26^). Each LC-SRM instrument method contained a list of targeted transitions which was derived from LC-QqQ-DDA-MS(/MS) spectrum libraries (described below).

In addition to LC-SRM analyses, the LC-QqQ system was operated as above but to perform shotgun DDA LC-MS(/MS) (MS^1^ scanning = 300–1500 m/z using Q3 to scan; the top 10 most intense precursors were selected for MS^2^). Two LC-QqQ-DDA-MS(/MS) analyses were run per sample so that the optimal collision energy settings for +2 and +3 precursor ions were used^26^.

PRM LC-MS was performed using an UltiMate 3000 nanoLC coupled to a QEHF mass spectrometer. The samples were pumped onto a trap column (Acclaim PepMap 100, C-18, 75 µm i.d., 2 cm length, Thermo Fisher Scientific Inc.), and analytes were resolved using an EASY-Spray column-ESI-tip cartridge (PepMap RSLC C18, 75 µm i.d., 50 cm length, 2 µm bead diameter with 100 A pores, Thermo Fisher Scientific Inc.). Analytes were separated using a 60 min linear gradient (2–30% Mobile Phase B; Mobile Phase A = 0.1% v/v FA; Mobile Phase B = 0.1% v/v FA in ACN; flow rate = 200 nl/min). Analytes were electrosprayed at 1.8 kV into the MS. Each MS^1^ scan (200–2000 m/z, Resolution = 60k) was followed by fourteen PRM scans (resolution = 60k, maximum isolation time = 110 ms, normalized collision energy = 27)^27–29^. Each LC-PRM instrument method contained a list of targeted precursor ions which was derived from LC-QEHF-DDA-MS(/MS) spectrum libraries (described below).

The LC-PRM analyses of the samples (Table 1) used a set of targeted precursor ions that were partitioned to make fourteen LC-PRM instrument method files. LC-MS scheduling was used, and “dynamic retention time” was set to ON. The BMDM-derived samples were analyzed in technical duplicate ( = 16 × 14 runs); the other samples (“B0-1”, “B0-2”, and “B0-3”) were each analyzed once ( = 3 × 14 runs). The resulting data were uploaded to Panorama Public^30^ (ProteomeXchange^31^ ID: PXD031697; 10.6069/44s8-9f68) (Table 2)^13^. LC-PRM was also performed using the heavy-labeled peptide standards alone to develop scheduled LC-PRM assays.

**Table 1:** Analyte per LC-MS injection of the samples used for absolute quantitation.

Sample	BMDM Replicate1 (μg)	BMDM Replicate2 (μg)	Firefly Luciferase (fmol)	Quantitated Peptide Stds. (fmol)	Total Mass (protein + peptide) (μg)	LC-MS Technical Replicates
B0-1			20	1	0.001339	1
B0-2			20	10	0.005598	1
B0-3			20	100	0.048189	1
B1-0	1		20	0	1.000865	2
B1-1	1		20	1	1.001339	2
B1-2	1		20	10	1.005598	2
B1-3	1		20	100	1.048189	2
B4-0		1	20	0	1.000865	2
B4-1		1	20	1	1.001339	2
B4-2		1	20	10	1.005598	2
B4-3		1	20	100	1.048189	2

Each sample preparation was scaled up 50-fold. BMDM, bone marrow-derived macrophage.

**Table 2:** Data records.

Filename	Location	Description
Manes TLR Chemotaxis 2022.sky.zip	10.6069/44s8-9f68	Skyline LC-PRM datafile (from the Table 1 samples), the spectral libraries, and the raw data (ProteomeXchange: PXD031697).
Targeted Proteins.xlsx	10.6069/44s8-9f68	Target proteins
Targeted Peptides.xlsx	10.6069/44s8-9f68	Target peptides
Transition List.xlsx	10.6069/44s8-9f68	Scheduled LC-PRM transitions
Skyline Raw Results.xlsx	10.6069/44s8-9f68	Raw data exported from the Skyline file of annotated mass spectrometry data (from LC-PRM of the Table 1 samples)
Final Quantitation Results.xlsx	10.6069/44s8-9f68	The final peptide- and protein-level quantitation results (from LC-PRM of the Table 1 samples)
Phosphoprotein Quantification.xlsx	10.6069/44s8-9f68	The final phosphoprotein quantitation results (from LC-PRM of the Table 1 samples)
Proteome-wide estimated abundances.xlsx	10.6069/44s8-9f68	Proteome-wide abundance estimates made using the RNA-seq data
Miscellaneous estimated abundances.xlsx	10.6069/44s8-9f68	Seventeen other abundance estimates (not made using the RNA-seq data; *e.g*., of lipids)

LC-PRM, liquid chromatography coupled to parallel reaction monitoring mass spectrometry; TLR, Toll-like receptor.

In addition to performing LC-PRM analyses, the LC-QEHF system was also used to perform shotgun and targeted DDA LC-MS(/MS) (MS^1^ range = 200–2000 m/z, MS^1^ resolution = 120k; the top 20 most intense precursors were selected for MS^2^; MS^2^ resolution = 60k, normalized collision energy = 27)^27–29^.

### LC-MS data analysis

The data analysis of the LC-QqQ-DDA-MS(/MS) spectra for the chemotaxis pathway has been described previously^2^. The data analysis of the TLR pathway shotgun LC-QqQ-DDA-MS(/MS) spectra followed our published protocol^11^. Specifically, these spectra were imported into Proteome Discoverer (v. 1.3.0.339 or 1.4.1.14, Thermo Fisher Scientific Inc.) and exported to a server running Mascot (v. 2.4.0 or 2.5.1, Matrix Science, London, UK) to perform database searching against a FASTA of the target peptides. The resulting Mascot PSMs were imported into Skyline^14^ (64-bit, v. 3.1.0.7382) to create spectral libraries for designing LC-SRM assays. LC-SRM spectra were analyzed using Skyline (64-bit, v. 3.1.0.7382) following our published protocol^11^.

Shotgun and targeted LC-QEHF-DDA-MS(/MS) was used to analyze the heavy-labeled peptide standards. The resulting spectra were analyzed using Proteome Discoverer (v. 2.2.0.388, Thermo Fisher Scientific Inc.) using Sequest HT and Mascot (v. 2.6.2, Matrix Science) to perform database searching against a FASTA of the target peptides. The static modifications were carbamidomethylation (C) and the heavy-isotope labels (K, R; despite these being static modifications, the unlabeled peptide forms were included by Mascot). The dynamic modifications were oxidation (H, M, W), acetylation (peptide N-term), deamidation (N, Q), Gln conversion to pyro-Glu (peptide N-term Q), and carbamidomethylation (peptide N-term). The spectra of the phosphopeptide standards were additionally searched for phosphorylation (S, T, Y) as a dynamic modification. The resulting Sequest and Mascot PSMs were imported into Skyline (64-bit, v. 4.2.0.19072) to create spectral libraries for designing Skyline LC-PRM assays. These spectral libraries contained 37 very low confidence PSMs of light (unlabeled) peptides, which could confound the downstream Skyline analyses. This was manually corrected using Sqlite Expert (Personal Edition x64 v. 5.3.4.459) to remove these 37 PSMs from the spectral libraries.

The LC-PRM spectra from the samples (Table 1) were imported into Skyline (64-bit, v. 19.1.0.193). For each precursor ion, the most intense product ions (maximum of 20) were annotated. The MS^1^ and MS^2^ mass error tolerance was 8 ppm (the range of the systematic mass error was 0 ppm to 4.1 ppm; the random error was approximately ± 1.5 ppm). The set of precursor ions had been partitioned into fourteen LC-PRM instrument methods, resulting in fourteen LC-PRM runs per Skyline “result”. Each transition was manually reviewed using criteria similar to the LC-SRM criteria that we described previously^11^, but adjusted for the higher mass accuracy of the LC-PRM data. The MS^1^ spectra (and any precursor ions in the MS^2^ spectra) were reviewed but not used for quantitation. The annotated Skyline results and the LC-QEHF-DDA-MS(/MS) spectral libraries were uploaded to Panorama Public^30^ (ProteomeXchange^31^ ID: PXD031697; 10.6069/44s8-9f68) (Table 2)^13^. The final table of transitions (“Transition List.xlsx”) and the transition-level quantitation data (“Skyline Raw Results.xlsx”) are available at Panorama Public^13^.

For each Skyline result and target peptide, the peptide light/heavy peak area ratio was calculated using the LC-MS peak areas of their common transitions, and it was equal to: (ΣLight)/(ΣHeavy). These values were multiplied by the heavy peptide fmol/injection values (Table 1), resulting in light peptide abundance values in units of fmol/injection. Using these values, and using the BMDM protein mass per injection values (Table 1), and using the median protein mass per BMDM cell value (determined using the BCA assays), target protein abundance values in units of copies/cell were calculated. From the samples that did not contain BMDM lysate (Table 1 “B0-1”, “B0-2”, and “B0-3”), the heavy peptide standard bleed-through (*i.e*., the peptide light/heavy ratio) was determined (the range was from 0% to 1%). The light peptide abundance values were corrected to remove bleed-through (if the bleed-through was >20% of the total light peptide abundance, the datum was discarded).

For each target peptide and for each replicate (technical and biological), a Quantitation Quality classification was made (equal to “Good” or “Maybe Poor”) to classify the quality of the pair (light and heavy) of peptide abundance values. It was equal to “Maybe Poor” if the number of quantitated common (light and heavy) transitions was ≤2, if the summed LC-PRM peak area (light or heavy) was at the low end the LC-PRM sensitivity limit (<10000), or if the peptide light/heavy ratio was extreme (heavy = 1 fmol and light >10 fmol, heavy = 10 fmol and light >500 fmol, or heavy = 100 fmol and light <2 fmol). Otherwise, the Quantitation Quality was equal to “Good”. For each target peptide and biological replicate, if there were two quantitation values with Quantitation Quality equal to “Good”, then any “Maybe Poor” quantitation values were discarded.

For each sample and target peptide, the geometric mean of the peptide abundance values was calculated across the technical replicates (including the stable isotope dilution series; thus, there was a maximum of 6 replicates). Relative absolute deviation from the mean (RADM) values were calculated; RADM is defined as ABS((X-M)/M) where X is a measurement of a quantity, M is the arithmetic mean of the measurements of the quantity, and ABS is the absolute value function. For each target peptide, the geometric mean was subsequently calculated across the two biological replicates. There were instances where multiple forms of the same peptide were quantified (*e.g*., unmodified and oxidized), and the lower abundance peptide forms were discarded. The seven discarded peptides were “HTDDEM[Ox]TGYVATR”, “LTPITYPQGLAM[Ox]AK”, “QKPITPETAEK”, “TAC[Carbam]TNFMM[Ox]TPYVVTR”, “TM[Ox]DAGC[Carbam]KPYM[Ox]APER”, “TM[Ox]DAGC[Carbam]KPYMAPER”, and “TMDAGC[Carbam]KPYM[Ox]APER” (“Ox” is oxidation and “Carbam” is carbamidomethyl). The remaining abundance values were summed across the peptide forms. A peptide was discarded if it was not unique to the target protein (unless this could be resolved using other unique peptides), or if the peptide identification was judged to be of low confidence^32^, or if a PTM (annotated in UniProt) confounded the quantification (“Peptide-to-Protein Consistency” column of “Final Quantitation Results.xlsx” available at Panorama Public^13^. For each target protein, the geometric mean was calculated across the target peptides to produce protein abundance values (in “Final Quantitation Results.xlsx” available at Panorama Public^13^). The targeted phosphoprotein data were analyzed as above but tabulated separately in “Phosphoprotein Quantification.xlsx” available at Panorama Public^13^.

### Proteome-wide abundance estimation

C57BL/6 J mice were used to produce BMDMs, and basal and LPS stimulated BMDMs were analyzed using RNA-seq^33^. The National Center for Biotechnology Information (NCBI) Gene Expression Omnibus (GEO) (https://www.ncbi.nlm.nih.gov/geo/) accession number for this RNA-seq dataset is GSE70510. The gene symbols were updated using the Mouse Genome Informatics online resource^34^ (http://www.informatics.jax.org/). For each sample and gene, a transcript per million (TPM) value was calculated^35^. A linear regression was performed using the basal BMDM Log_10_-transformed TPM values and the LC-PRM Log_10_-transformed copy/cell values. The resulting equation was used to estimate protein copies/cell values for the entire BMDM proteome (tabulated in “Proteome-wide estimated abundances.xlsx” available at Panorama Public^13^). Seventeen additional abundance estimates were made (*e.g*., of lipids) (tabulated in “Miscellaneous estimated abundances.xlsx” available at Panorama Public^13^).

## Data Records

The LC-QEHF-DDA-MS(/MS) RAW files, the LC-QEHF-MS/MS spectral libraries, the LC-PRM RAW files, and the annotated Skyline dataset were deposited in the Panorama Public targeted proteomics data repository^30^ (ProteomeXchange^31^ ID: PXD031697; 10.6069/44s8-9f68) (Table 2)^13^. The supplemental tables were deposited in Panorama Public under the “Supplementary Data” tab (upper right). On the “Panorama Dashboard” tab (upper right) of the webpage is a description of the project and a link to download the annotated Skyline datafile (“Manes TLR Chemotaxis 2022.sky.zip”). This file can be directly opened in Skyline (it does not need to be unzipped first). Opening it in Skyline will open the annotated LC-PRM datafiles (“Manes TLR Chemotaxis 2022.sky”, “Manes TLR Chemotaxis 2022.sky.view”, and “Manes TLR Chemotaxis 2022.skyd”), the spectral library datafiles (“QStdsQEHF_PD20190531A2.blib”, “QStdsQEHF_PD20190531B2.blib”, and “QStdsQEHF_PD20190531C.blib”), and the indexed retention time datafile (iRT_2019-09-23_001.irtdb) in a single step. Alternatively, “Manes TLR Chemotaxis 2022.sky.zip” can be unzipped using file compression software and the files can be opened individually.

On the “Raw Data” tab (upper right) of the webpage is a folder tree of the raw datafiles. The “SpecLibr” folder contains the datafiles used to create the three BLIB spectral library datafiles. The contents of this folder are described in detail in a Microsoft Excel file (“_Spectral library data analyses description.xlsx”). The nineteen other folders of the folder tree are named after the samples in Table 1 (“a” and “b” indicates the technical (LC-PRM) replicate). Each of these folders contain fourteen RAW datafiles of raw LC-PRM data (one RAW file for each of the fourteen LC-PRM instrument files). Each of these nineteen folders corresponds to a Skyline “result” within the annotated Skyline datafile (“Manes TLR Chemotaxis 2022.sky.zip”).

## Technical Validation

### Overview of the results

In this investigation, targeted proteomics was used to measure the absolute abundance (copies/cell) of mouse macrophage proteins of the TLR4 and chemotaxis pathways. After developing the LC-PRM assays, eleven samples were analyzed (Table 1) resulting in the data described in Table 2.

**Fig. 1:**
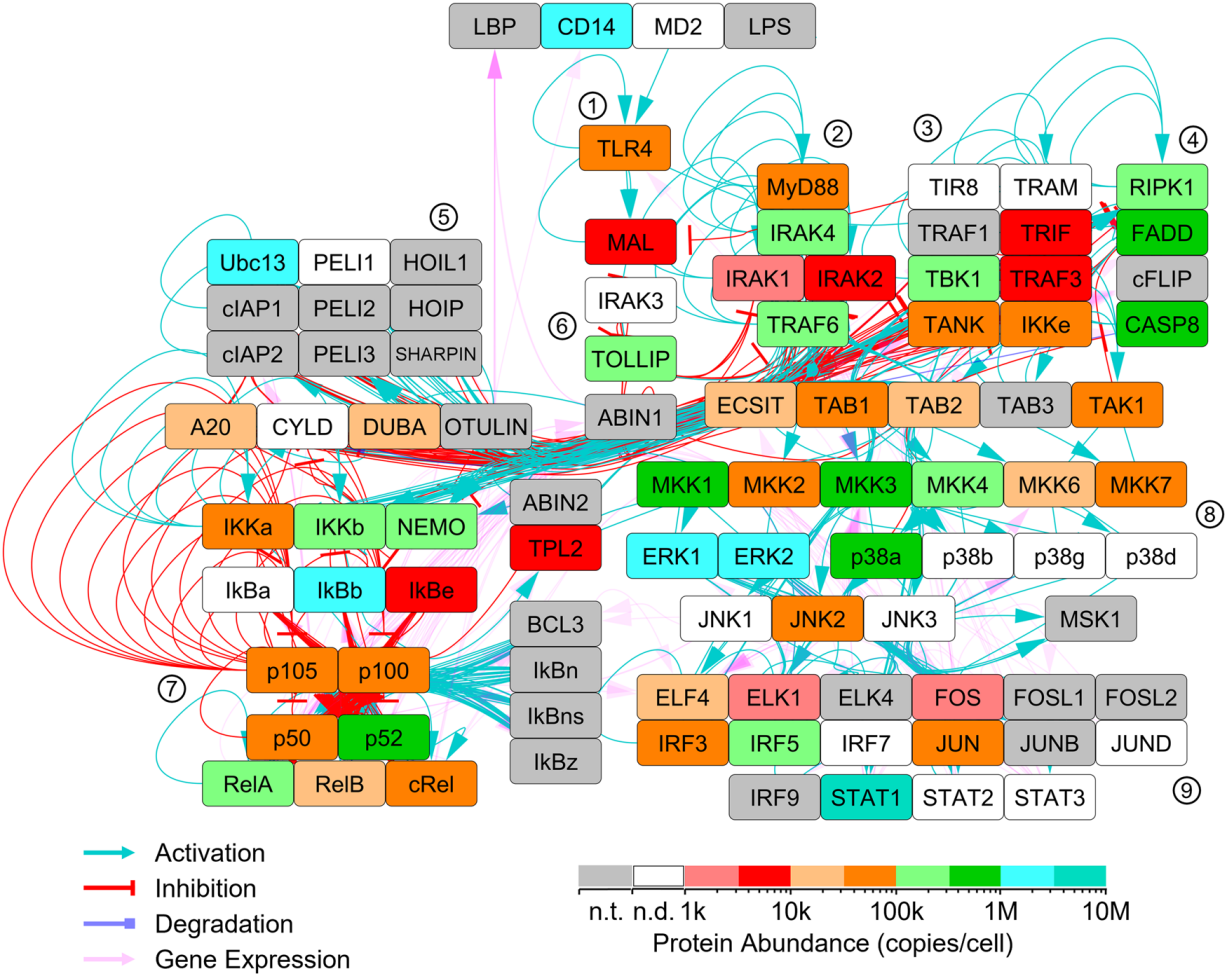
Quantitation of core TLR4 pathway proteins within mouse BMDMs. LC-PRM assays were performed to quantitate proteins of the core TLR4 pathway. Some proteins were targeted but were not detected (white nodes), possibly because their abundance was below the limit of detection of the LC-MS. Selected clusters of nodes are indicated by encircled numbers: 1. TLR4 receptors, 2. myddosome signaling complex, 3. triffosome signaling complex, 4. programmed cell death, 5. K63 and M1 polyubiquitination, 6. negative regulators, 7. NFκB pathway, 8. MAP kinase signaling, 9. transcription factors. This signaling network is described in detail in our earlier article^1^. n.d., not detected. n.t., not targeted.

**Fig. 2:**
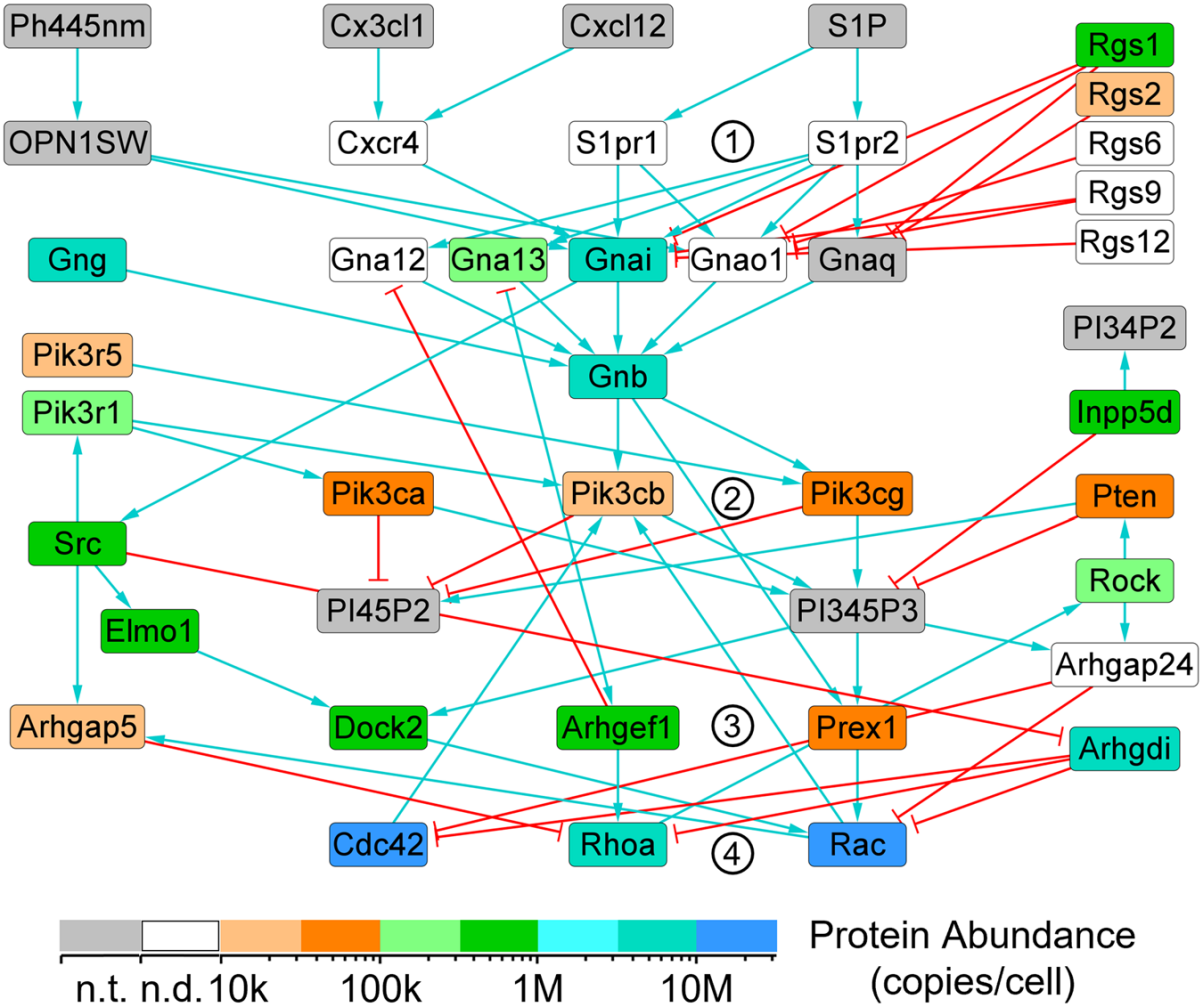
Quantitation of chemotaxis signaling pathway proteins within mouse BMDMs. LC-PRM assays were performed to quantitate proteins of the chemotaxis signaling pathway. Some proteins were targeted but were not detected (white nodes), possibly because their abundance was below the limit of detection of the LC-MS. Selected clusters of nodes are indicated by encircled numbers: 1. G-protein coupled receptors, 2. phosphatidylinositol 4,5-bisphosphate 3-kinases, 3. guanine nucleotide exchange factors, and 4. small GTPases. This signaling network is described in detail in our earlier article^2^. n.d., not detected. n.t., not targeted.

The target protein abundance values (copies/cell) within mouse BMDMs are tabulated in “Final Quantitation Results.xlsx” available at Panorama Public^13^. These data were mapped onto the core TLR4 signaling pathway (Fig. 1) and the chemotaxis signaling pathway (Fig. 2). These networks are described in detail in our earlier articles^1,2^. Some proteins of these pathways have not yet been targeted. Of the proteins that were targeted, most were successfully quantitated. The proteins that were targeted but not successfully quantitated might have had abundance values that were below the limit of detection of the LC-PRM analyses. Additional rounds of LC-PRM assay development are underway to further investigate the mouse macrophage TLR and chemotaxis pathways.

**Fig. 3:**
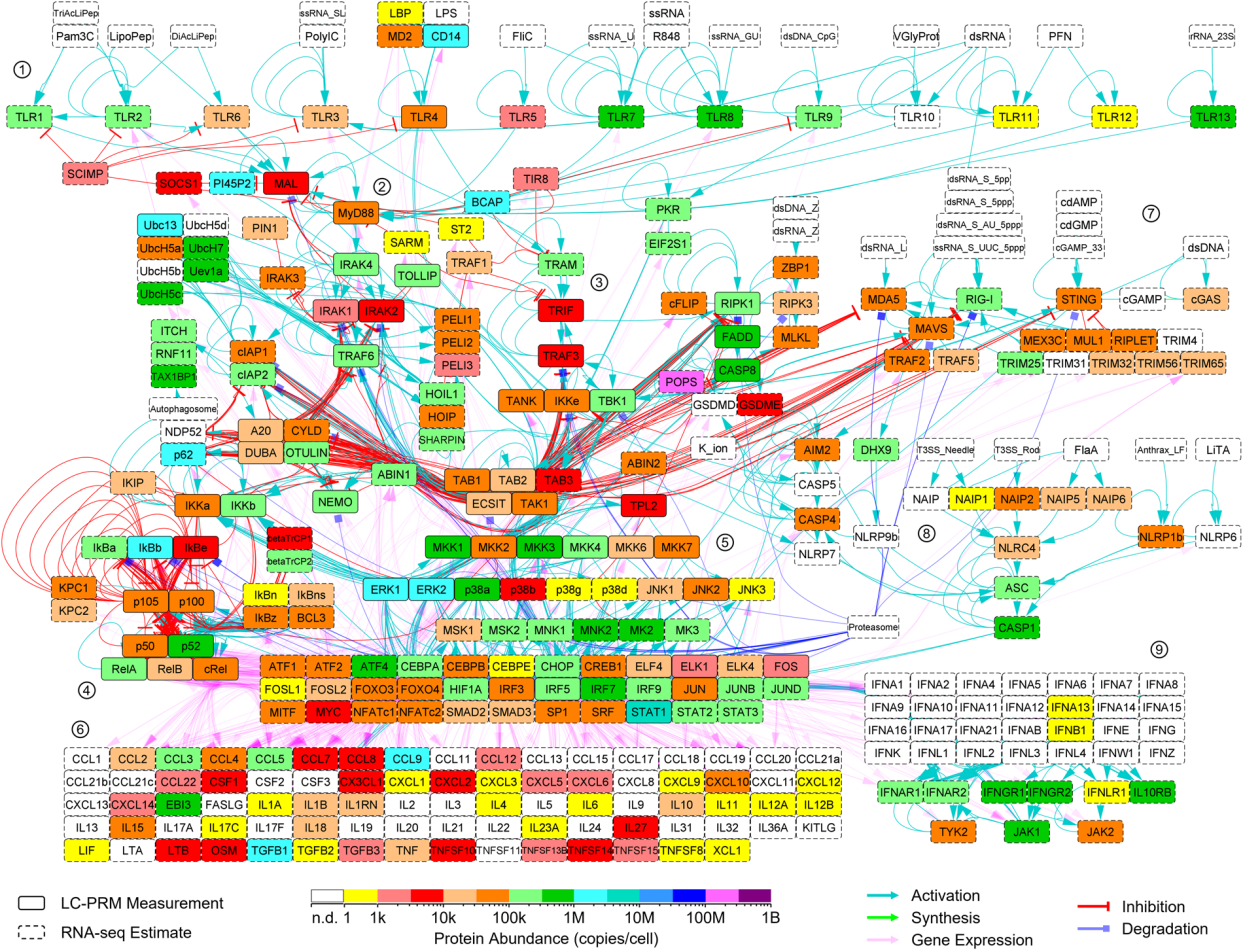
Quantitation of PRR pathway proteins within mouse BMDMs. LC-PRM assays were performed to quantitate proteins of the core TLR4 pathway. In the absence of an LC-PRM measurement, an RNA-seq estimate was used. Selected clusters of nodes are indicated by encircled numbers: 1. TLR receptors, 2. myddosome signaling complex, 3. triffosome signaling complex, 4. NFκB pathway, 5. MAP kinase signaling, 6. gene expression of cytokines, 7. STING and MAVS signaling, 8. inflammasomes, 9. interferon pathway. This signaling network is described in detail in our earlier article^1^. n.d., not detected.

**Fig. 4:**
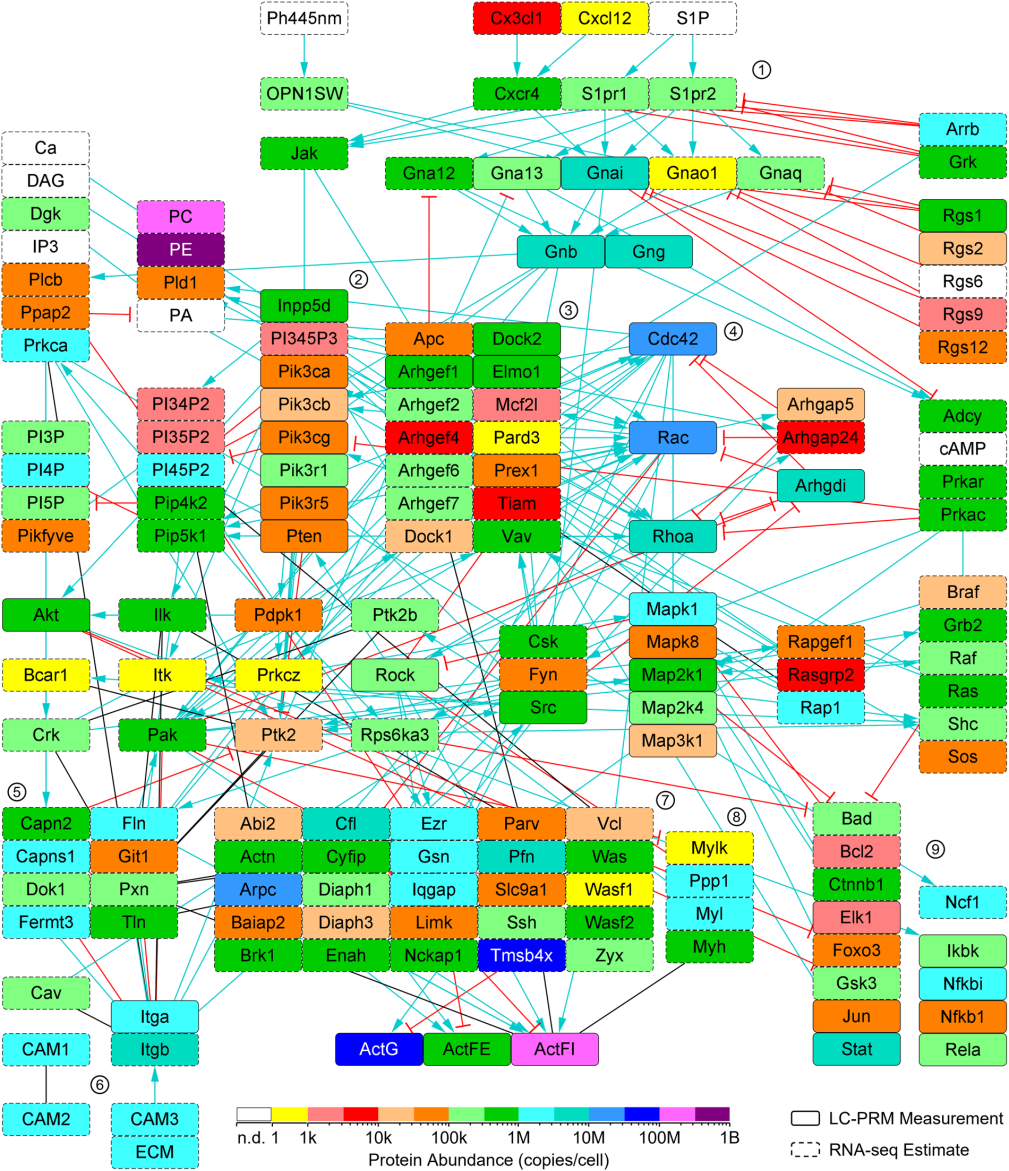
Quantitation of chemotaxis pathway proteins within mouse BMDMs. LC-PRM assays were performed to quantitate proteins of the signaling component of the chemotaxis pathway. In the absence of an LC-PRM measurement, an RNA-seq estimate was used. Selected clusters of nodes are indicated by encircled numbers: 1. G-protein coupled receptors, 2. phosphatidylinositol 4,5-bisphosphate 3-kinases, 3. guanine nucleotide exchange factors, 4. small GTPases, 5. focal adhesions, 6. integrin and cell adhesion molecules, 7. actin cytoskeleton, 8. actomyosin, and 9. transcription factors. This network is described in detail in our earlier article^2^. n.d., not detected.

A previously published mouse BMDM RNA-seq dataset^33^ was found to be moderately correlated with the target protein absolute abundance dataset, and these two datasets were used to estimate protein abundance values for the entire BMDM proteome (tabulated in “Proteome-wide estimated abundances.xlsx” available at Panorama Public^13^). The LC-PRM measurements and RNA-seq estimates were mapped onto a PRR signaling network (Fig. 3) and the full chemotaxis pathway (Fig. 4). These networks are described in detail in our earlier articles^1,2^.

### Quantitation of TLR4 and chemotaxis pathway proteins within mouse macrophages

In this investigation, LC-PRM assays were developed and used to quantitate proteins of the core TLR4 and chemotaxis signaling pathways within mouse BMDMs. Protein quantitation using a pair of differently quantitated internal peptide standards (AAA versus JPT-QTag UV absorption) was investigated, and the two methods resulted in similar protein quantitation (Figure S1). Two biological replicates of BMDMs were prepared (using one mouse each). To confirm that the TLR pathway of the cells was not unintentionally stimulated, the cells were analyzed using a multiplexed western blot against phospho-JNK and phospho-ERK1/2. The two phosphoproteins were much lower in abundance in the basal state cells compared to the LPS stimulated cells (Figure S2).

Eleven samples were prepared for LC-PRM, and eight of the samples were LC-PRM analyzed twice (Table 1). The raw transition-level abundance values are tabulated in the file “Skyline Raw Results.xlsx”) available at Panorama Public^13^. For each target peptide, the heavy-labeled peptide standard bleed-through was calculated (Figure S3). The LC-PRM data were adjusted to account for the bleed-through.

Variance due to technical variation was examined (Figure S4). The CV values were ~15% for most of the peptides. At the low end of the abundance range, the CV values were higher, indicating that these measurements were probably below the LC-PRM limit of quantitation. Variance due to biological variation was examined (Figure S5). The CV values were ~10% for most of the peptides. At the low end of the abundance range, the CV values were higher, again indicating that these measurements were probably below the LC-PRM limit of quantitation.

Overall, the assays developed during this investigation conform to the “Tier 2” (non-clinical) criteria described in a broadly accepted guidance for targeted LC-MS assays^36^. The Tier 2 precision requirement is “typically <20–35% CV achieved”. For each target peptide, the CV was calculated across the replicates (technical and biological), and 200 of the 214 peptide assays (93%) had a CV value that was <35%. The other Tier 2 requirements were fully satisfied by the peptide assays.

Variance across the peptide targets was examined (Figure S6). The variation was classified as “consistent”, “semi-consistent”, or “inconsistent”, and most of the results were “consistent”. Our peptide target selection criteria were designed to avoid artifacts that would interfere with protein quantitation, but it is likely that the semi-consistent and inconsistent variance was caused by currently unknown issues such as unreported RNA splicing, posttranslational modification, and/or chemical artifacts. The accuracy and precision of the assays might be different for different samples. To resolve these issues and improve quantitation of these proteins, additional target peptide assays will need to be developed. This dataset was produced to parameterize models of the TLR and chemotaxis pathways. For some of the target proteins, the measurements across some of the target peptides were inconsistent, so we are continuing to develop target peptide assays to perform additional measurements. The final peptide- and protein-level quantitation results were tabulated in “Final Quantitation Results.xlsx” available at Panorama Public^13^. For completeness, the small amount of phosphoprotein data was tabulated in “Phosphoprotein Quantification.xlsx” available at Panorama Public^13^.

**Fig. 5:**
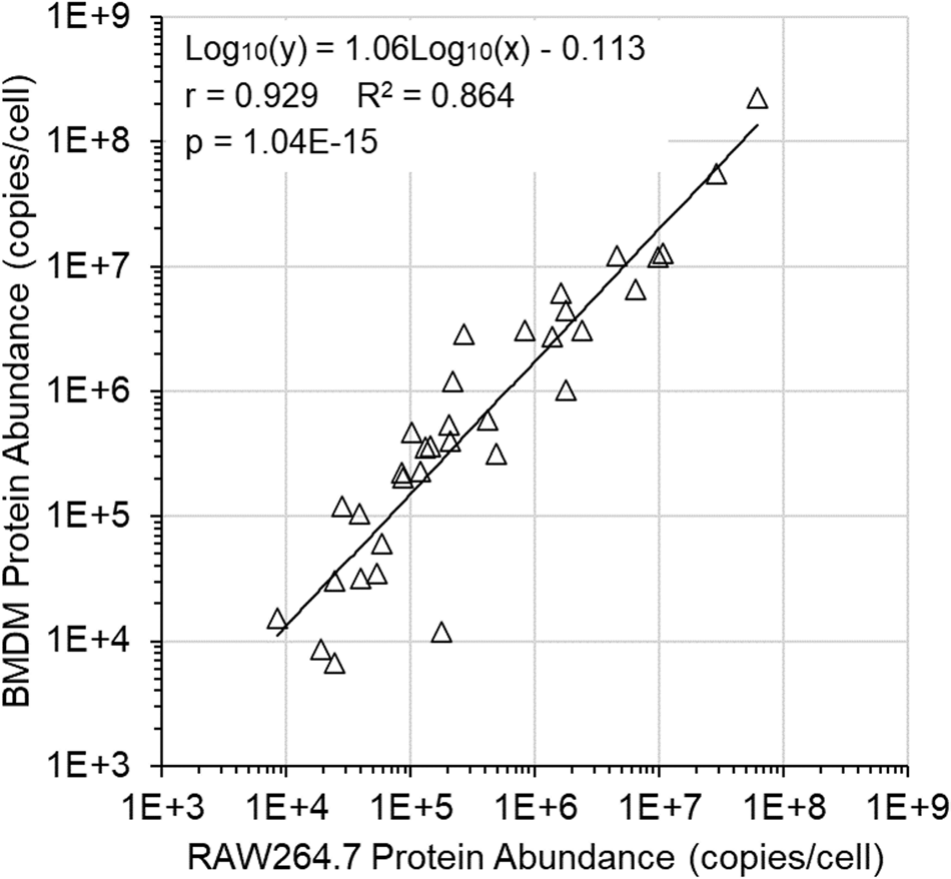
Strong correlation between two related datasets. We used LC-SRM to measure the absolute abundance of chemotaxis pathway proteins within RAW264.7 mouse monocyte/macrophage cells^2^. These quantitation values correlated strongly with the BMDM LC-PRM data reported here. Note that the linear regression is not along the diagonal but is skewed upward. For each protein, a ratio was calculated: [BMDM copies/cell]/[RAW264.7 copies/cell]. The geometric mean of these ratios was 1.63. Additionally, we measured that the RAW264.7 and BMDM protein mass was 136 pg/cell and 230 pg/cell (respectively), a 1.69-fold difference. This indicates that our LC-MS measurements were highly precise.

To investigate the precision of our protein quantitation results, we compared these data to an LC-SRM dataset from our earlier publication^2^. These earlier experiments used the RAW264.7 mouse monocyte/macrophage cell line to investigate the chemotaxis pathway. The two datasets were found to be strongly correlated (Fig. 5). The average BMDM contained 1.63-fold the target protein abundance of the average RAW264.7 cell. Additionally, the average BMDM contained 1.69-fold the protein mass of the average RAW264.7 cell. The strong correlation between the two datasets and the near-equality of the two ratios (1.63 versus 1.69) is strong evidence that our targeted LC-MS quantitation measurements were highly precise across the two cell types and the two LC-MS methods.

The LC-PRM protein quantitation results were mapped onto the core TLR4 signaling pathway (Fig. 1). This network is described in detail in our earlier article^1^. Some proteins of the pathway have not yet been targeted. Most of the proteins that were targeted were successfully quantitated. Likewise, the LC-PRM data were mapped onto the chemotaxis signaling pathway (Fig. 2). This network is described in detail in our earlier article^2^. The targeted proteins that were not successfully quantitated might have had abundance values that were below the limit of detection of the LC-MS. Additional rounds of LC-PRM assay development are underway to further investigate the mouse macrophage TLR and chemotaxis pathways.

### Proteome-wide abundance estimation

We and others have previously reported moderate correlation between mRNA and protein absolute abundance values for numerous cell/tissue types: *Escherichia coli*^37,38^, *Saccharomyces cerevisiae*^38–44^, *Schizosaccharomyces pombe*^45^, *Caenorhabditis elegans*^46^, the RAW264.7 (mouse monocyte/macrophage) cell line^2^, the NIH/3T3 (mouse fibroblast) cell line^47^, seven mouse brain cell types^48^, the HeLa (human epithelial) cell line^49,50^, twelve human tissues^51,52^, fourteen human tissues^53^, and eleven human tissues and nine human cell lines^54^. In addition, transcript abundance was found to be the major determinant of protein abundance in mouse dendritic cells^55^.

**Fig. 6:**
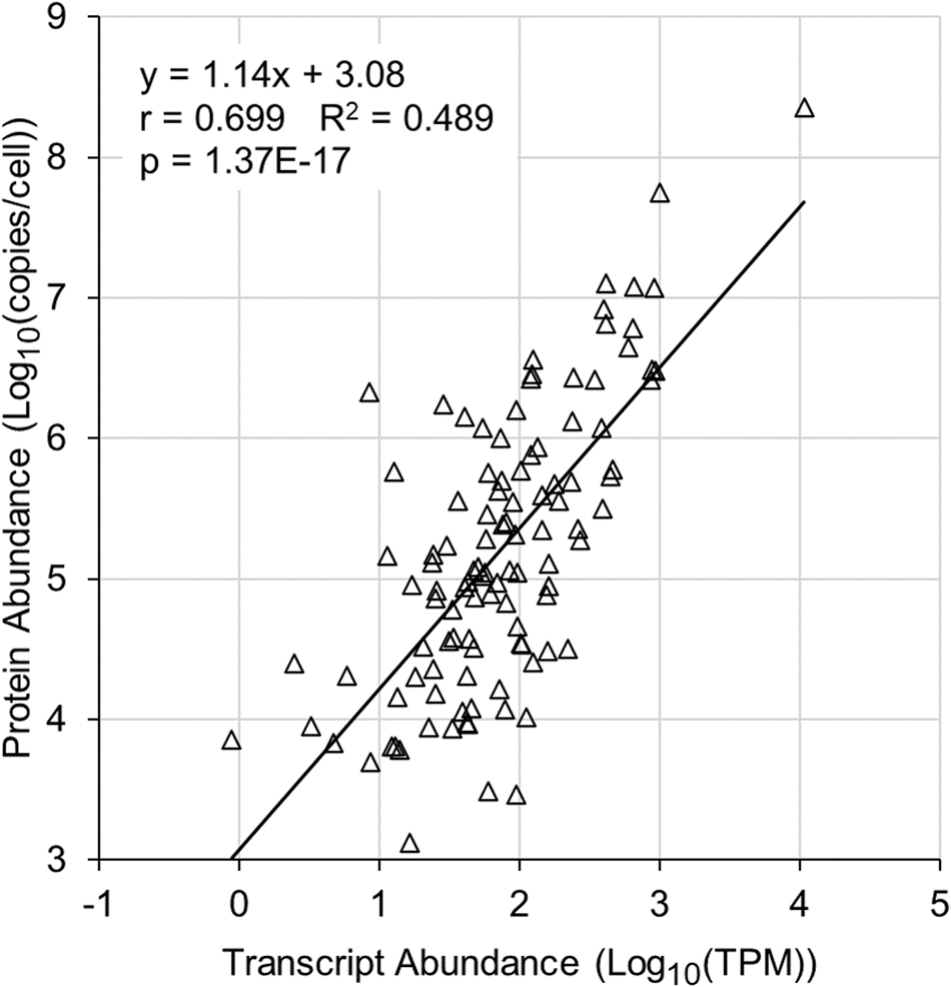
BMDM transcript and protein abundance values were moderately correlated. Log_10_-transformed abundance values of BMDM transcripts^33^ and proteins were analyzed using a linear regression, and a moderate correlation was found. For each protein, a ratio was calculated: [modeled protein copies/cell]/[measured protein copies/cell] (if < 1, the reciprocal was used). The mean, geometric mean, and median of these ratios was 7.51, 3.70, and 3.01, respectively. This linear regression was used to estimate protein abundance values for the entire BMDM proteome. These results are similar to our previously reported results using RAW264.7 cells^2^.

In our earlier investigation, we performed a linear regression analysis of a RAW264.7 RNA-seq dataset with a RAW264.7 targeted proteomics dataset^2^. The two datasets were found to be correlated (r = 0.8827, R^2^ = 0.7792). Using the LC-PRM dataset and a previously published BMDM RNA-seq dataset^33^, we again performed a linear regression analysis. The two datasets were found to be moderately correlated (Fig. 6). The linear regression was used to estimate protein abundance values for the entire BMDM proteome (tabulated in “Proteome-wide estimated abundances.xlsx” available at Panorama Public^13^). The median error (modeled-to-measured abundance ratio) of the estimated target protein abundance values was 3.01-fold. It should be noted that while half of the error values were 3.01-fold or less, the error value was more than 10-fold for some of the genes. Seventeen of the estimates were not made using the RNA-seq dataset (*e.g*., of lipids) (tabulated in “Miscellaneous estimated abundances.xlsx” available at Panorama Public^13^).

The LC-PRM measurements and RNA-seq estimates were mapped onto a PRR signaling network (Fig. 3). This network is described in detail in our earlier article^1^. Likewise, the LC-PRM measurements and RNA-seq estimates were mapped onto the full chemotaxis pathway (Fig. 4). This network is described in detail in our earlier article^2^.

## Supplementary information

Supplementary figures
