# Supplementary material for: Absolute protein quantitation of the mouse macrophage Toll-like receptor and chemotaxis pathways
Source: Sci Data. 2022 Aug 12;9:491. doi: 10.1038/s41597-022-01612-y (PMC9374760; doi:10.1038/s41597-022-01612-y)
Supplement: Supplementary file 1 — Supplementary figures [file 41597_2022_1612_MOESM1_ESM.pdf]

Absolute protein quantitation of the mouse macrophage Toll-like receptor and chemotaxis pathways

Nathan P. Manes, Jessica M. Calzola, Pauline R. Kaplan, Iain D. C. Fraser,
Ronald N. Germain, Martin Meier-Schellersheim, Aleksandra Nita-Lazar

Scientific Data 2022 Supplemental Figures

Figure S1	Protein Quantitation using Heavy-Labeled Peptide Standards that were Quantitated using either Amino Acid Analysis or JPT-QTag UV Absorption	2
Figure S2	Western blot to confirm that the untreated BMDMs did not display characteristics of TLR-stimulation..	3
Figure S3	Heavy Isotope-Labeled Peptide Standard Bleed-Through..............................	4
Figure S4	Variance Across the Technical Replicates..	5
Figure S5	Variance Across the Biological Replicates...	7
Figure S6	Variance Across the Target Peptides...	9

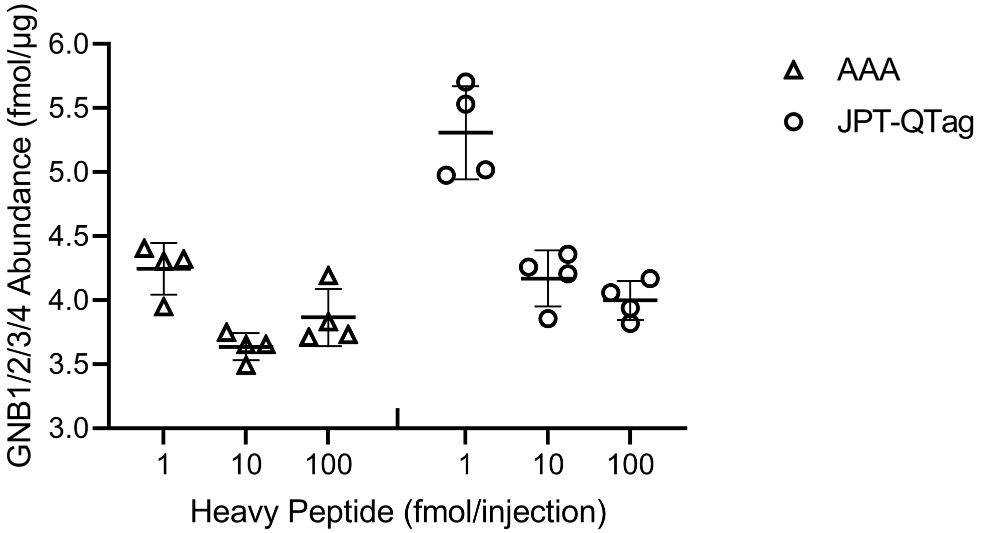

Figure S1. Protein Quantitation using Heavy-Labeled Peptide Standards that were Quantitated using either Amino Acid Analysis or JPT-QTag UV Absorption.

The accuracy of protein quantitation was tested using a pair of peptide standards that were quantitated using different methods: amino acid analysis (AAA) or UV absorption of the trypsin-cleavable carboxy-terminal JPT-QTag [Schnatbaum et al 2020 Proteomics 20:2000007]. The target protein was $G_β$ (specifically, GNB1 + GNB2 + GNB3 + GNB4), and it was targeted using the target peptide "LLVSASQDGK". Two peptide standards were acquired from JPT Peptide Technologies GmbH: LLVSASQDGK[$^{13}C_6$,$^{15}N_2$] was quantitated using AAA, and LLVSASQDGK[$^{13}C_6$,$^{15}N_2$].[JPT-QTag] was quantitated using UV absorption ("." indicates a trypsin cleavage site). A preparation immortalized mouse macrophage (IMM) cell lysate was split into 32 tubes (each tube received 20 µg (protein mass)). For each sample, 0, 20, 200, or 2000 fmol of one of the two peptide standards was added. The samples were reduced using DTT, alkylated using iodoacetamide, digested using trypsin, and desalted using OMIX C-18 SPE tips (Agilent, Santa Clara, CA). The samples were analyzed using LC-QEHF-PRM (1 µg (peptide mass) per injection), and the resulting data were manually annotated using Skyline (64-bit, v. 19.1.0.193). IMM $G_β$ was quantitated in units of fmol per µg (IMM protein mass) (values are mean +/- SD).

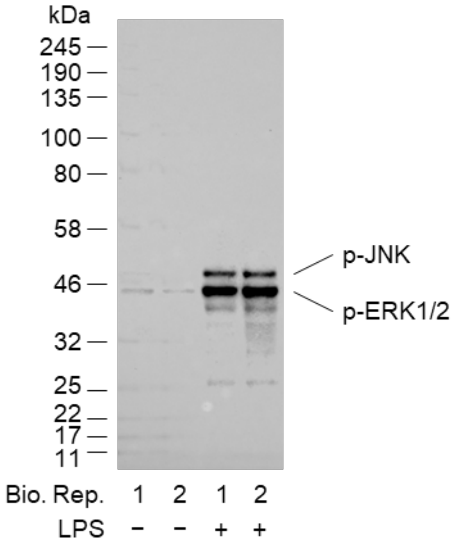

Figure S2. Western blot to confirm that the untreated BMDMs did not display characteristics of TLR-stimulation.
Two biological replicates of BMDMs were prepared (each using one mouse). For each biological replicate, one dish was unstimulated, and another dish was stimulated for 30 min with LPS (specifically, 10 nM Kdo2-Lipid A). The cells were analyzed using a multiplexed western blot against phospho-JNK and phospho-ERK1/2.

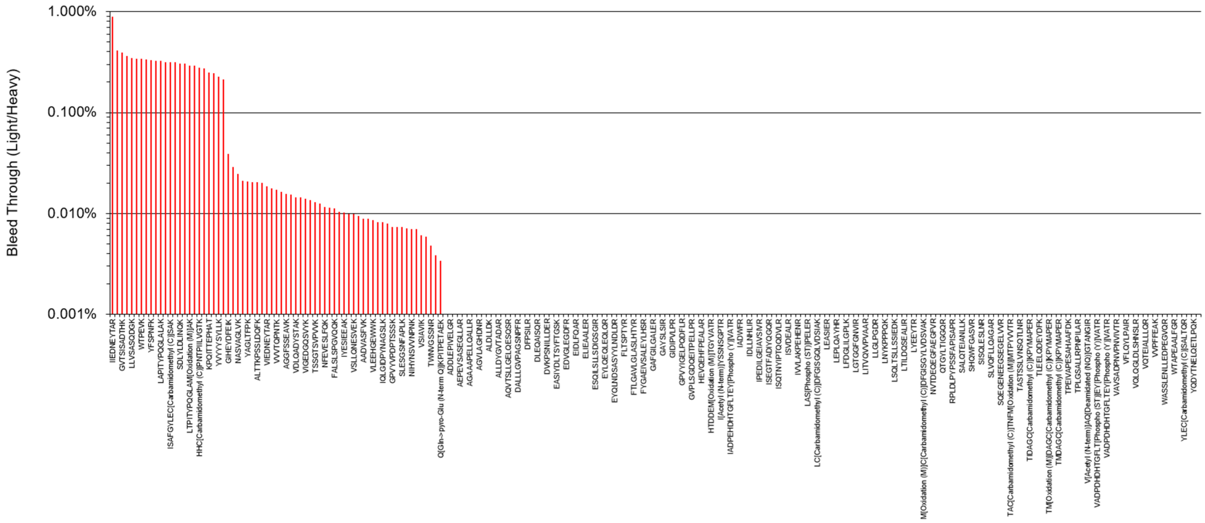

Figure S3. Heavy Isotope-Labeled Peptide Standard Bleed-Through.
The heavy isotope-labeled, purified, quantified peptide standards (alone) were analyzed using LC-PRM using both the light and heavy precursor ions and transitions. Detectable bleed-through was detected in ~30% of the peptide standards and ranged from 0% to 1%. Note that only ~50% of the peptide sequences are visible due to space limitations along the horizontal axis.

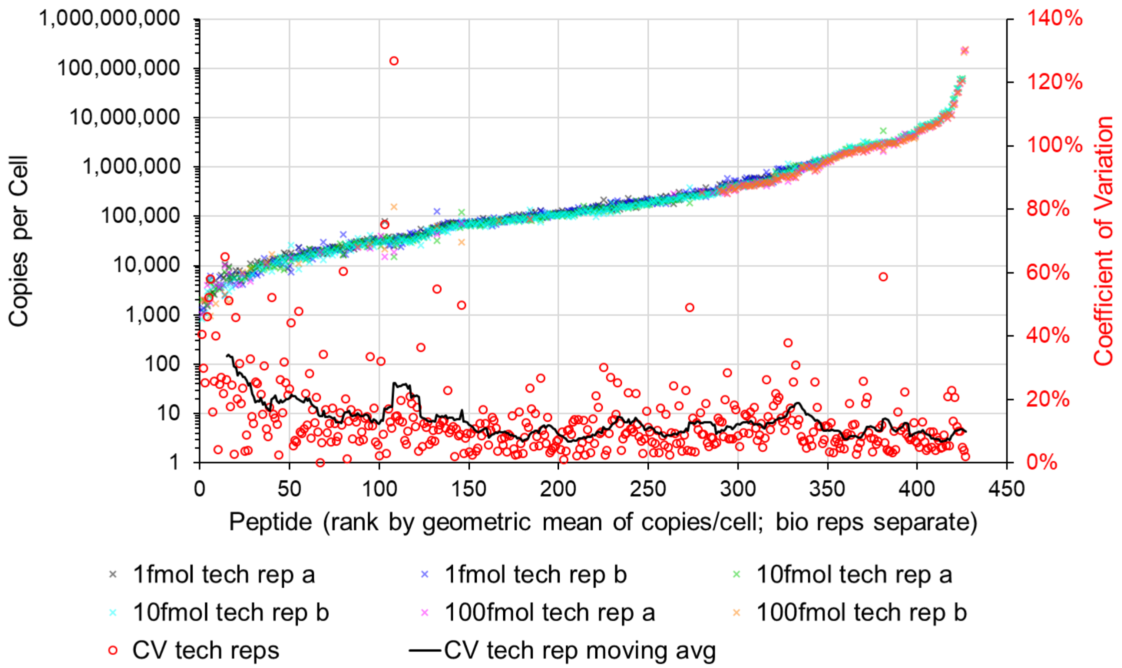
Copies per Cell
1,000,000,000
100,000,000
10,000,000
1,000,000
100,000
10,000
1,000
100
10
1
0
50
100
150
200
250
300
350
400
450
140%
120%
100%
80%
60%
40%
20%
0%
Coefficient of Variation
Peptide (rank by geometric mean of copies/cell; bio reps separate)
1fmol tech rep a
1fmol tech rep b
10fmol tech rep a
10fmol tech rep b
100fmol tech rep a
100fmol tech rep b
CV tech reps
CV tech rep moving avg

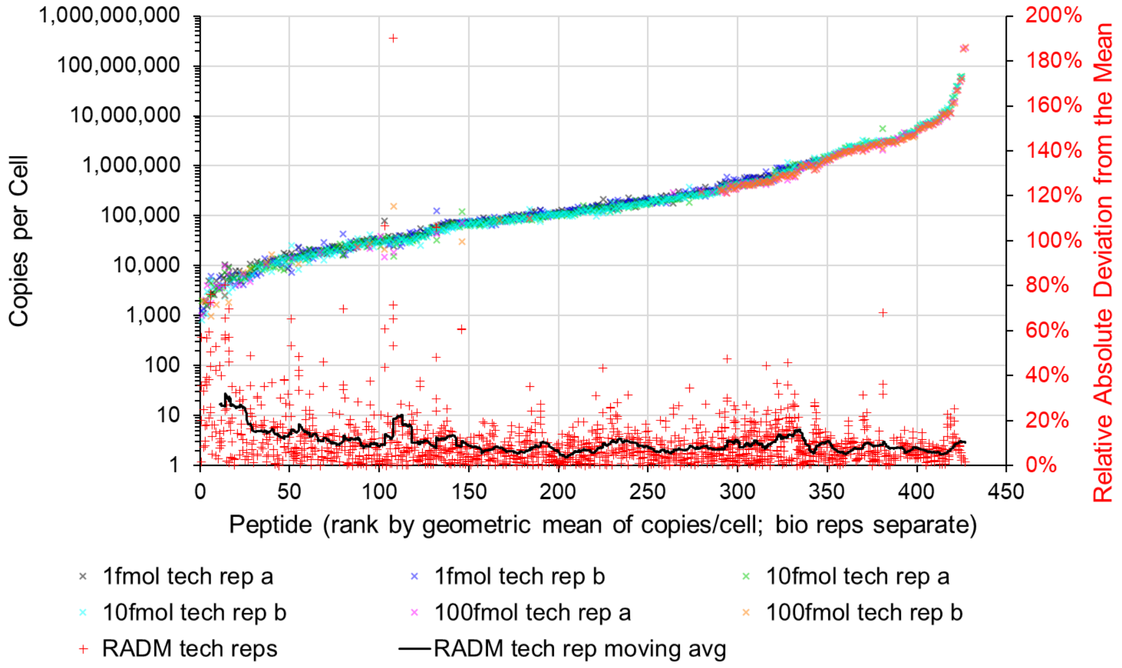
Copies per Cell
1,000,000,000
100,000,000
10,000,000
1,000,000
100,000
10,000
1,000
100
10
1
0
50
100
150
200
250
300
350
400
450
200%
180%
160%
140%
120%
100%
80%
60%
40%
20%
0%
Relative Absolute Deviation from the Mean
Peptide (rank by geometric mean of copies/cell; bio reps separate)
1fmol tech rep a
1fmol tech rep b
10fmol tech rep a
10fmol tech rep b
100fmol tech rep a
100fmol tech rep b
RADM tech reps
RADM tech rep moving avg

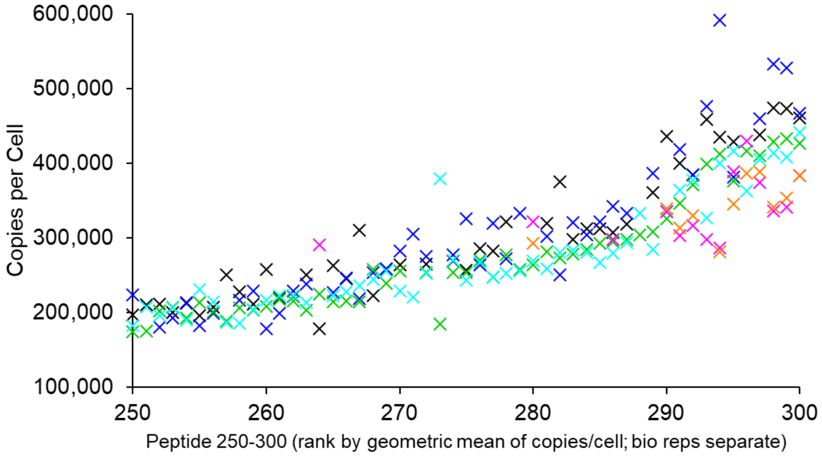

Figure S4. Variance Across the Technical Replicates.
Top: Each target peptide was used to measure the corresponding target protein copies per cell (left y-axis). For each peptide and biological replicate, the geometric mean of the abundance values was calculated across the technical replicates (including both the LC-MS replicate runs "a" and "b", and the heavy isotope dilution series; thus, a maximum of six values). These geometric mean values were ranked (x-axis). Additionally, the coefficient of variation was calculated across the technical replicates (right y-axis). The two biological replicates were kept separate (*i.e.*, a peptide might be present twice along the x-axis). **Middle:** Same as the top graph but using the relative absolute deviation from the mean. **Bottom:** A subset of the data with the y-axis scaled normally (*i.e.*, not Log_{10}-transformed).

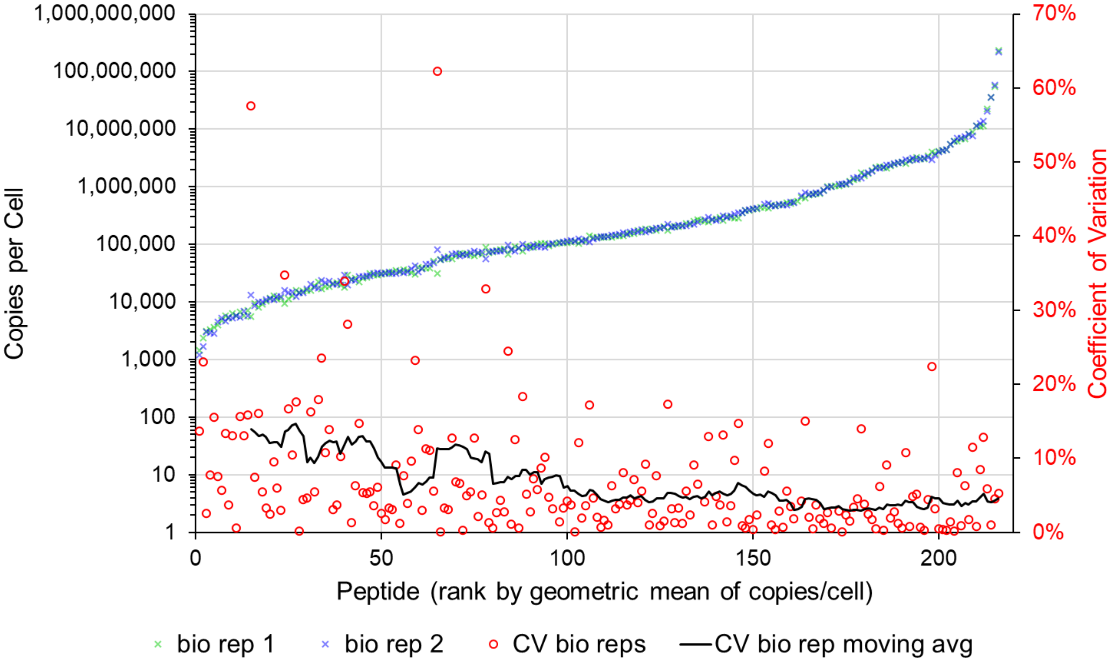

1,000,000,000
100,000,000
10,000,000
1,000,000
100,000
10,000
1,000
100
10
1
70%
60%
50%
40%
30%
20%
10%
0%
0
50
100
150
200
Copies per Cell
Coefficient of Variation
Peptide (rank by geometric mean of copies/cell)
bio rep 1
bio rep 2
CV bio reps
CV bio rep moving avg

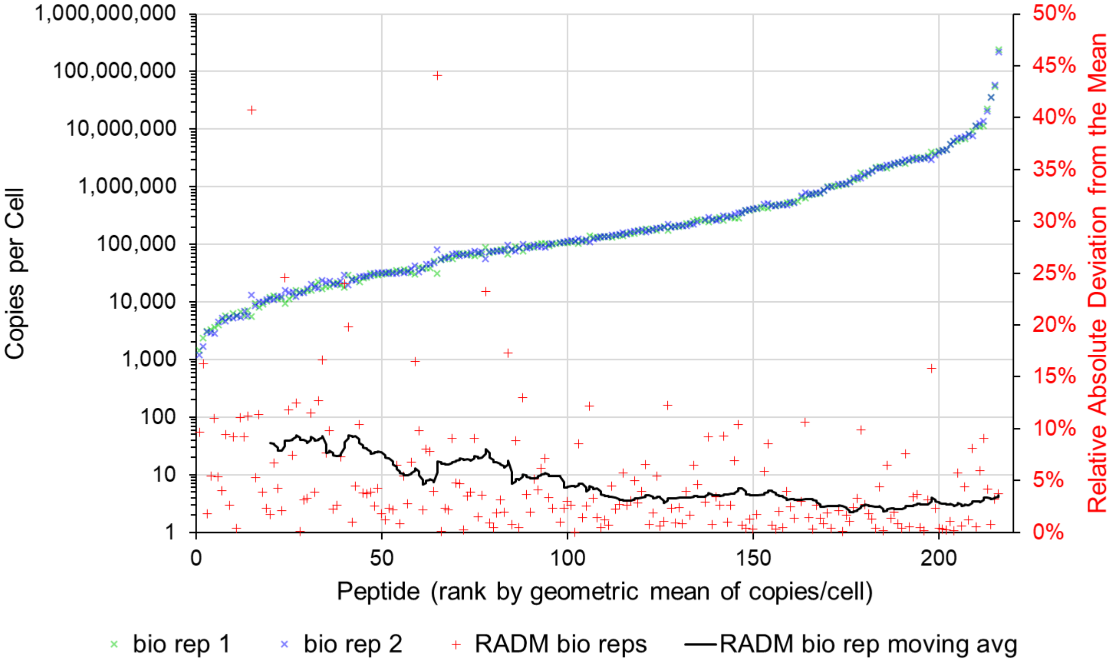

1,000,000,000
100,000,000
10,000,000
1,000,000
100,000
10,000
1,000
100
10
1
50%
45%
40%
35%
30%
25%
20%
15%
10%
5%
0%
0
50
100
150
200
Copies per Cell
Relative Absolute Deviation from the Mean
Peptide (rank by geometric mean of copies/cell)
bio rep 1
bio rep 2
RADM bio reps
RADM bio rep moving avg

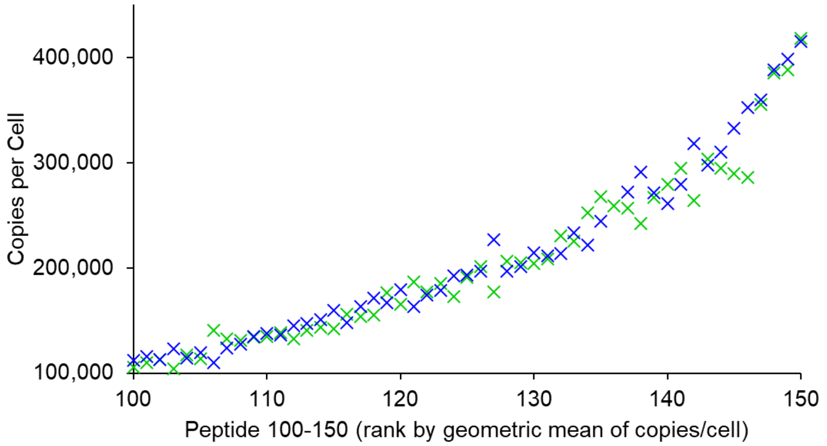

Figure S5. Variance Across the Biological Replicates.
Top: Each target peptide was used to measure the corresponding target protein copies per cell. For each peptide and biological replicate, the geometric mean of the abundance values was calculated across the technical replicates (including both the LC-MS replicate runs "a" and "b", and the heavy isotope dilution series; thus, a maximum of six values) (left y-axis). For each peptide, the geometric mean of the abundance values was calculated across the two biological replicates. These values were ranked (x-axis). Additionally, the coefficient of variation was calculated across the two biological replicates (right y-axis). For each peptide, the abundance ratio was calculated ([bio rep 1] / [bio rep 2]), and the mean of these ratios was 0.9896, the median of these ratios was 0.9874, and the geometric mean of these ratios was 0.9787. **Middle:** Same as the top graph but using the relative absolute deviation from the mean. **Bottom:** A subset of the data with the y-axis scaled normally (*i.e.*, not Log_{10}-transformed).

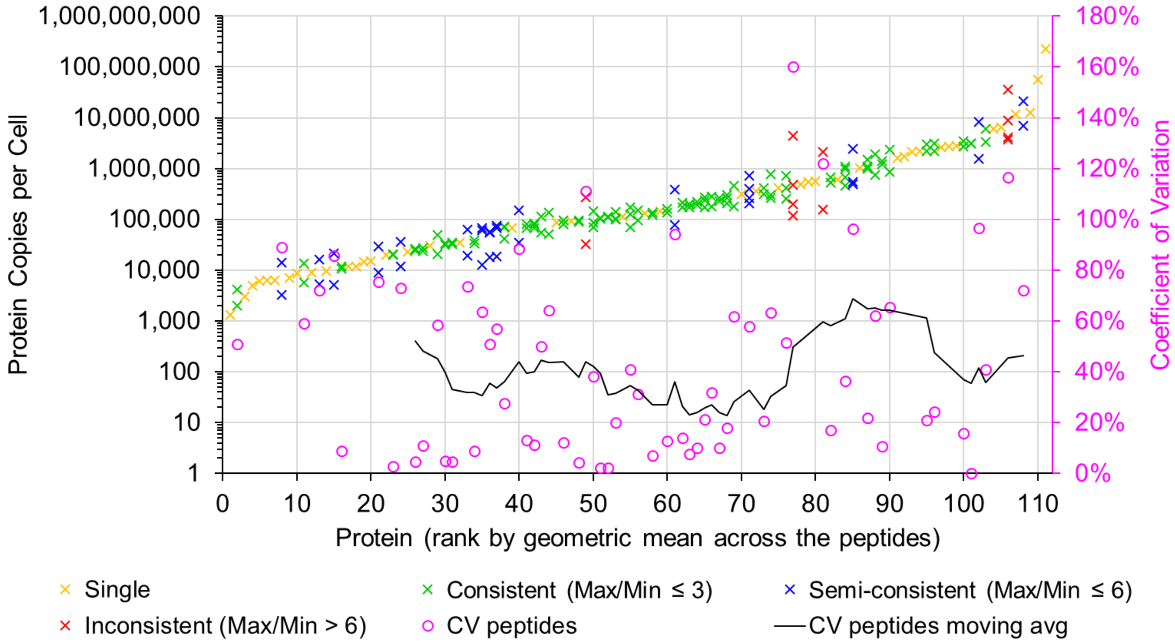
Protein Copies per Cell
1,000,000,000
100,000,000
10,000,000
1,000,000
100,000
10,000
1,000
100
10
1
180%
160%
140%
120%
100%
80%
60%
40%
20%
0%
Coefficient of Variation
0
10
20
30
40
50
60
70
80
90
100
110
Protein (rank by geometric mean across the peptides)
Single
Consistent (Max/Min ≤ 3)
Semi-consistent (Max/Min ≤ 6)
Inconsistent (Max/Min > 6)
CV peptides
CV peptides moving avg

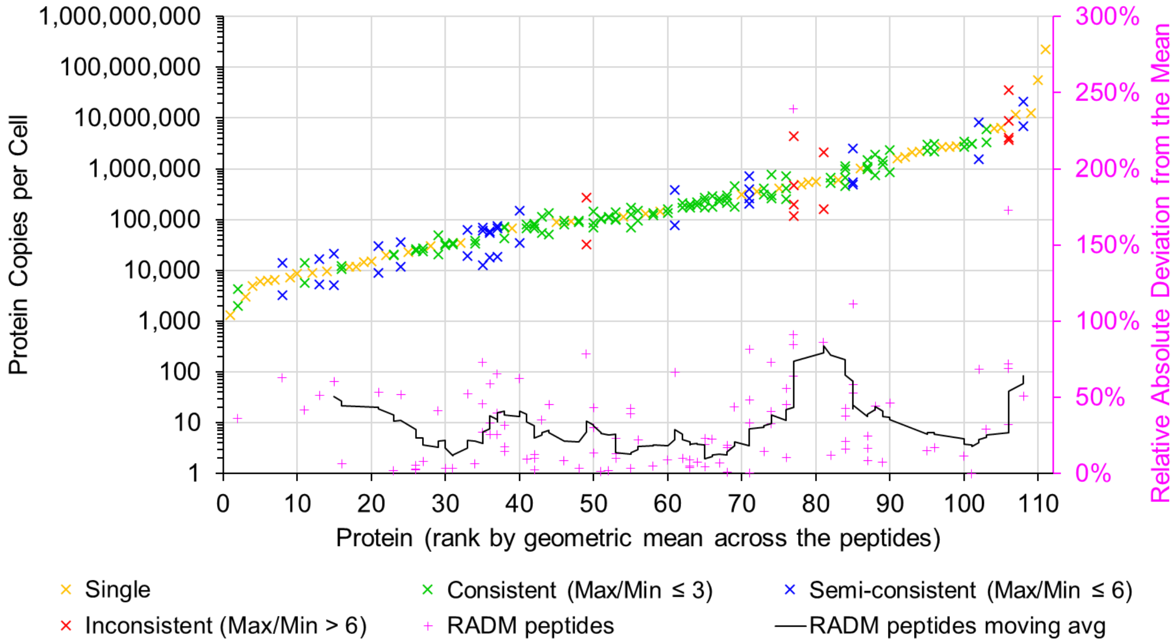
Protein Copies per Cell
1,000,000,000
100,000,000
10,000,000
1,000,000
100,000
10,000
1,000
100
10
1
300%
250%
200%
150%
100%
50%
0%
Relative Absolute Deviation from the Mean
0
10
20
30
40
50
60
70
80
90
100
110
Protein (rank by geometric mean across the peptides)
Single
Consistent (Max/Min ≤ 3)
Semi-consistent (Max/Min ≤ 6)
Inconsistent (Max/Min > 6)
RADM peptides
RADM peptides moving avg

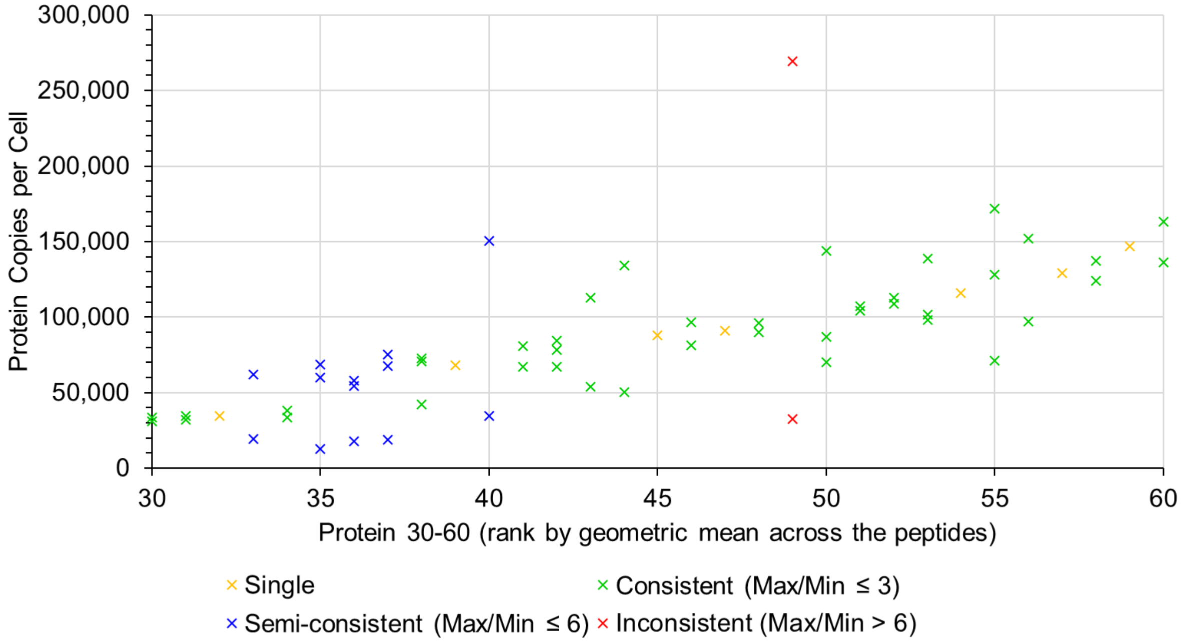

Figure S6. Variance Across the Target Peptides.
Top: For each target peptide, the geometric mean of the peptide abundance values was calculated across the technical replicates, and then across the biological replicates (left y-axis). For each target protein, the geometric mean of the peptide abundance values was calculated across the target peptides. These values were ranked (x-axis). Additionally, the coefficient of variation was calculated across the target peptides (right y-axis). Each target peptide was classified as "Single" (*i.e.*, the sole target peptide for the corresponding target protein), "Consistent" (the maximum/minimum peptide abundance value ratio was ≤ 3 for the target protein), "Semi-consistent" (3 < max/min ≤ 6), or "Inconsistent" (max/min > 6). **Middle:** Same as the top graph but using the relative absolute deviation from the mean. **Bottom:** A subset of the data with the y-axis scaled normally (*i.e.*, not Log_{10}-transformed).
